# Integrated Measure of PRogram Element SuStainability in Childcare Settings (IMPRESS-C): development and psychometric evaluation of a measure of sustainability determinants in the early childhood education and care setting

**DOI:** 10.1186/s13012-024-01372-w

**Published:** 2024-06-20

**Authors:** Adam Shoesmith, Nicole Nathan, Melanie Lum, Serene Yoong, Erin Nolan, Luke Wolfenden, Rachel C. Shelton, Brittany Cooper, Cassandra Lane, Alice Grady, Noor Imad, Edward Riley-Gibson, Nicole McCarthy, Nicole Pearson, Alix Hall

**Affiliations:** 1 School of Medicine and Public Health, University of Newcastle, Newcastle, NSW 2308 Australia; 2 Hunter New England Population Health, Hunter New England Local Health District, Newcastle, NSW 2287 Australia; 3 Hunter Medical Research Institute, New Lambton Heights, NSW 2305 Australia; 4 Faculty of Health, School of Health and Social Development, Global Centre for Preventive Health and Nutrition, Institute for Health Transformation, Deakin University, Geelong, VIC 3220 Australia; 5 School of Health Sciences, Department of Nursing and Allied Health, Swinburne University of Technology, Hawthorn, VIC 3122 Australia; 6 Department of Sociomedical Sciences, Mailman School of Public Health, Columbia University, New York, NY 10032 USA; 7 Department of Human Development, Washington State University, Pullman, WA 99164 USA; 8 National Centre of Implementation Science, University of Newcastle, Callaghan, NSW 2308 Australia

**Keywords:** Sustainability, Sustainment, Measurement, Development, Reliability, Validity, Early childhood education and care

## Abstract

**Background:**

There is a need for valid and reliable measures of determinants of sustainability of public health interventions in early childhood education and care (ECEC) settings. This study aimed to develop and evaluate the psychometric and pragmatic properties of such a measure – the Integrated Measure of PRogram Element SuStainability in Childcare Settings (IMPRESS-C).

**Methods:**

We undertook a two-phase process guided by the COnsensus-based Standards for the selection of health status Measurement INstruments checklist (COSMIN) and Psychometric and Pragmatic Evidence Rating Scale (PAPERS). Phase 1 involved measure development; i.e., determining items and scales through an iterative process and assessment of face and content validity. Phase 2 involved the evaluation of psychometric and pragmatic properties. The 29-item measure completed by service executives (directors and nominated supervisors) was embedded in a larger survey from a national sample of Australian ECEC services assessing their implementation of nutrition and physical activity programs. Structural validity, concurrent validity, known groups validity, internal consistency, floor and ceiling effects, norms, and pragmatic qualities of the measure were assessed according to the PAPERS criteria.

**Results:**

The final measure contained 26 items, with respondents reporting how strongly they agreed or disagreed on a five-point Likert scale. Phase 1 assessments confirmed the relevance, and face and content validity of the scale. In Phase 2, we obtained 482 completed surveys, of which 84% (*n =* 405) completed the entire measure across 405 ECEC settings (one executive per service). Three of the four fit indices for the confirmatory factor analysis met the pre-specified criteria (SRMR = 0.056, CFI = 0.993, RMSEA = 0.067) indicating ‘good’ structural validity. The IMPRESS-C illustrated: ‘good’ internal consistency, with Cronbach’s alpha values from 0.53 to 0.92; ‘emerging’ concurrent validity; ‘poor’ known groups validity; ‘good’ norms; and ‘good’ overall pragmatic qualities (cost, readability, length, and assessor burden).

**Conclusions:**

The IMPRESS-C possesses strong psychometric and pragmatic qualities for assessing service executive-level perceptions of determinants influencing sustainment of public health interventions within ECEC settings. To achieve a full range of perspectives in this setting, future work should be directed to also develop and test measures of sustainability determinants at the implementer level (e.g., among individual educators and staff).

**Supplementary Information:**

The online version contains supplementary material available at 10.1186/s13012-024-01372-w.

Contributions to the literature

- There is a need to develop valid, reliable, and pragmatic measures of sustainability determinants designed and evaluated for ECEC settings. This study aimed to develop and evaluate the psychometric and pragmatic properties of the first known measure of sustainability determinants in the ECEC setting at the executive level.
- This measure of sustainability determinants illustrated ‘good’ structural validity, ‘good’ internal consistency, ‘emerging’ concurrent validity, ‘good’ norms, and ‘good’ pragmatic qualities (cost, readability, length and assessor burden).
- These findings enhance the existing evidence base by providing a measure to assess key determinants that shape intervention sustainment from the perspective of service executives. This will enable an accurate and tailored approach to developing strategies to support intervention sustainment within ECEC settings.

## Background

There are a multitude of effective evidence-based interventions (EBI) that are delivered in community settings to reduce risk factors for chronic disease and improve population health [1–5]. However, implementation of these EBIs, and their effects, often attenuate once initial implementation support or external funding is withdrawn [6, 7]. This has found to be the case for a range of interventions across community, education and clinical settings [6–10]. The sustained implementation of EBIs is important to ensure that they continue to yield positive effects among patients and populations, and that the considerable investment required to achieve successful initial implementation is not wasted [9].

Sustainability has been defined as *‘after a defined period of time, the program, clinical intervention, and/or implementation strategies continue to be delivered and/or individual behaviour change (i.e., clinician, patient) is maintained; the program and individual behaviour change may evolve or adapt while continuing to produce benefits for individuals/systems’* [11]. An important step in understanding and addressing EBI sustainability is the accurate identification and assessment of the characteristics, or determinants, that impact sustainability [10, 12, 13]. This enables researchers, policymakers and practitioners to develop strategies that address priority determinants to support EBI sustainability. Systematic reviews investigating the multi-level determinants of EBI sustainability have identified a number of factors perceived by stakeholders to be influential in the context of early educational settings [7, 14, 15]. The determinants most frequently identified in these settings include: the availability of equipment, resources and facilities, continued executive or leadership support, staff turnover, alignment with existing external policies, and workplace socio-cultural factors [7, 14, 15].

There are also a number of theories and frameworks that propose how these determinants interact and function to shape sustainability [9, 16–18]. One such framework, the Integrated Sustainability Framework by Shelton and colleagues, was informed by empirical evidence and comprehensively identifies and theorises the interactions between determinants found to be influential to sustainment across a range of interventions delivered in “real world” clinical and community settings [9]. Influential determinants are organised into five domains including Outer Contextual Factors, Inner Contextual Factors, Processes, Intervention Characteristics, and Implementer/Population Characteristics [9]. This framework provides a useful structure for understanding, assessing and addressing the determinants of program sustainability. Although there are validated measures available that cover aspects of these framework domains and constructs [19], there are currently no formal validated quantitative measures that align with, and comprehensively cover this framework, hindering the framework’s utility to better understand the determinants and mechanisms of EBI sustainability.

Improving measurement of key implementation constructs and their determinants, including those pertaining to sustainability, is a priority for the field [20]. These are often assessed using self-report measures completed by key informants within specific settings (i.e., executive leadership and practitioners involved in EBI delivery). To identify the accuracy and validity of self-report measures, it is important to undertake thorough psychometric evaluations. Specifically, such measures should comprehensively cover the intended construct [21], assess reliability [22], as well as important pragmatic qualities, including the measure’s ease of access, use, scoring, and interpretation [23, 24]. To minimise misunderstanding and increase measurement accuracy, it is also important to ensure the correct determinants are measured from relevant perspectives (i.e., specific questions asked for different roles – executive vs. implementer level) [20, 25]. For example, determinants relating to higher-level organisational structures and processes that occur (e.g., funding allocation or external partnership support) should be answered by executives within the organisation (i.e., Directors, Managers, Supervisors, Leaders) who have in-depth knowledge of such structures and processes [25].

High-quality systematic reviews have been conducted examining available measures of sustainability (as an outcome) and sustainability determinants across a range of settings, their psychometric and pragmatic properties, and how they have been empirically used [20, 26, 27]. The most recent of these conducted by Hall and colleagues in 2022 [20], provided researchers with a comprehensive guide to identify where robust and suitable measures exist and provide practical guidance to end-users in selecting the most relevant measure for their setting [20]. The review included 223 articles representing 28 individual measures, of which two assessed sustainability as an outcome [28, 29], 25 assessed sustainability determinants, and only one explicitly assessed both [30]. The review used the Psychometric and Pragmatic Evidence Rating Scale (PAPERS) to assess the psychometric and pragmatic qualities of each measure [24, 31]. The Provider Report of Sustainment Scale (PRESS) measure [28] was found to be the most psychometrically robust and pragmatic measure of sustainability, however this measure assesses sustainability as an outcome (i.e., continued delivery of an EBI), and does not cover important determinants found to influence EBI delivery. The highest rating measure of sustainability determinants was the School-wide Universal Behaviour Sustainability Index-School Teams (SUBSIST) [32], however this is limited to evaluating a specific EBI – School-wide Positive Behavioral Interventions and Supports within schools, and is not appropriate when considering other EBIs in other settings. Further, whilst the Clinical Sustainability Assessment Tool (CSAT) [33] and Sustainment Measurement System Scale (SMSS) [30] illustrated favourable psychometric and pragmatic qualities compared to other measures of sustainability determinants, it was recommended that the CSAT be considered for use when assessing sustainability determinants in clinical settings, and the SMSS for evaluating prevention programs and initiatives that have been or are currently funded by Substance Abuse and Mental Health Services Administration.

Evidently, whilst a range of measures have been identified, most have only been used once or a small number of times [28, 30, 34–36], are limited to a specific EBI [32, 34, 37–39], or have variable psychometric and pragmatic quality [29, 40–42], illustrating limited standardisation and quality in measurement [20, 27]. Furthermore, no measure of sustainability determinants has been developed and psychometrically evaluated within some key settings for the implementation of interventions focusing on children, such as early childhood education and care (ECEC) settings (i.e., formal, paid or government‐subsidised services that offer care for children six years and under, prior to commencing compulsory schooling [5]). The ECEC setting is a key target setting for implementing and sustaining effective chronic disease prevention interventions as they provide access to a large proportion of children for prolonged periods at critical stages in their development [43]. While there have been numerous EBIs in the ECEC setting found to be effective in improving child physical activity and healthy eating [4, 5], little is known about the determinants that impact their sustainability, with only two previous studies actively investigating sustainability determinants in the ECEC setting [15, 45].

As the organisational structure, curriculum, staffing, type of interventions and delivery processes differ considerably across settings [44], including ECEC, so too are the factors likely contributing to EBI sustainability [15, 45]. This presents a growing need to investigate these factors to help support intervention sustainability in the ECEC setting. However, systematic review evidence illustrates that in comparison to other settings, such as schools, there are no known validated measures of sustainability determinants available in this setting [20]. Therefore, the development and consistent use of large-scale, setting-specific, psychometrically robust, and pragmatic measures of sustainability determinants in ECEC services is required, to improve our understanding of what influences EBI sustainability in this setting. Therefore this study aimed to:

1. Develop a self-report measure – Integrated Measure of PRogram Element SuStainability in Childcare Settings (IMPRESS-C) designed to assess determinants of sustainability of evidence-based public health interventions in ECEC settings from the perspective of the service executive.
2. Evaluate psychometric properties of the measure, including: structural validity; concurrent validity; known groups validity; internal consistency; floor and ceiling effects; and norms.
3. Assess pragmatic properties of the measure, including: cost; readability; training; length; ease of access; and interpretation.

## Methods

The processes for development and psychometric evaluation of the IMPRESS-C were guided by the COnsensus-based Standards for the selection of health status Measurement INstruments (COSMIN) checklist [46], and Psychometric and Pragmatic Evidence Rating Scale (PAPERS) [24, 31]. These are regarded as gold standard guidelines for measure development [46], and assessment of measure psychometric and pragmatic properties [24, 31]. As recommended, the development of this measure was conducted over two phases: *Phase 1: item development, face and content validity; and Phase 2: psychometric and pragmatic evaluation*.

### Phase 1: item development, face and content validity

#### Item development

Measure domains and items were informed by constructs from the Integrated Sustainability Framework [9] and systematic review evidence of determinants of sustainability in the ECEC setting [15, 45]. The Integrated Sustainability Framework was selected as it: (i) highlights key multi-level determinants that the emerging evidence suggests are important for facilitating intervention sustainability across a range of types of settings, including ECEC services [9]; (ii) can help identify and organise determinants that may be important in facilitating sustainability of an EBI; and (iii) provides clear definitions for how determinants can be categorised into framework domains [15]. The framework organises sustainability determinants into the following five domains: Outer Contextual Factors, Inner Contextual Factors, Processes, Characteristics of the Interventionist and Population, and Characteristics of the Intervention [9] (see Table 1).

**Table 1:** Integrated Sustainability Framework domains with corresponding factors covered, definitions, and examples of application

Domain	Factors covered^a^	Factor definition^b^	Examples of application within ECEC settings^c^
**Outer Contextual Factors**	• Policy and legislation • Sociopolitical context • Funding environment • Leadership • Values, priorities, needs • Community ownership	**Sociopolitical context:** The external landscape, including existing policies and regulations, guidelines, and mandates that pose implications on the sustainment of evidence-based interventions (EBIs). This may also include sociocontextual norms or policies that are discriminatory or stigmatising	External attention, e.g., from government, institutions, or agencies on programmes or interventions, national certification, or government policies
		**Funding environment and availability:** The funding landscape, including nature, stability, scope, diversity, and length of the funding environment	Provision of funding support from external sources, e.g., government or non-governmental organisations
		**External partnerships and leadership/environmental support:** Receiving external support through networks and partnerships (e.g., through engagement or resource exchange with academic and health organisations and community partners), support, commitment, and involvement from national leadership	Partnership with a university or health organisation who provides support (e.g., through the provision of resources or training from a local Area Health Service)
		**Values, needs, priorities:** The extent to which an EBI or topic is regarded as a national priority or fits with national, state, or local organisational priorities, needs, and values	Governmental policies and priorities, e.g., Federal Government prioritisation of obesity within Australia
**Inner Contextual Factors**	• Funding/resources • Leadership/support • Climate/culture • Staffing turnover • Structural characteristics • Capacity • Champion • Polices (alignment) • Mission	**Programme champions:** Individuals who have strong influences on the behaviours, attitudes, and norms of their colleagues/peers and promote the ongoing delivery of EBIs	Having an effective school champion (e.g., classroom teacher, physical education teacher, stage coordinator, or school executive) who leads and is responsible for driving the ongoing delivery of a health-promoting programme within schools
		**Organisational leadership/support:** Support from those within the organisation who have formal responsibility for leading, organising, and overseeing the programme	Support from school principals, executives, and other teachers
		**Organisational readiness/resources:** The level of resources and support internally to facilitate the ongoing delivery of a programme (e.g., space, money, funding, time)	Allocated in-school funding for a health-promoting programme provided by the principal; or in-school access to resources, e.g., adequate equipment and programme materials or adequate space
		**Organisational stability:** Staff attrition and turnover of space, organisation, staffing, or leadership	Determining the impact of staff turnover, e.g., teaching staff, principals, and school champions on the ongoing delivery of a health-promoting programme in schools
**Processes**	• Partnership/engagement • Training/support/supervision • Fidelity • Adaptation • Planning • Team/board functioning • Programme evaluation/data • Communication • Technical assistance • Capacity building	**Partnership/engagement:** Processes to directly and actively engage with key stakeholders (e.g., community board, role modelling, and networking)	Advisory groups and meetings with P&C committees and other stakeholders to provide updates about a health-promoting programme in schools
		**Training/supervision/support:** Processes related to implementation strategies (e.g., formal education or training, formal supervision processes, or other forms of support)	Provision of booster workshops and training to up-skill teachers or school champions to facilitate the ongoing delivery of a health-promoting programme in schools
		**Programme evaluation/data:** Collection of data and assessment or feedback to inform programme planning and decisions	Conducting process evaluation surveys with participants involved, e.g., principals, teachers, parents, or students to inform what programme strategies were effective or ineffective and make improvements to facilitate the ongoing delivery of a health-promoting programme
		**Adaptation:** Processes in place to actively and systematically guide adaptation of a policy or programme	Implementing a plan for adaptation to enable the alteration of a health-promoting programme as required, e.g., introducing wet weather plans and plans for casual teachers. This also includes the ability to adapt a programme based on factoring including climate or geographical location, e.g., implementing a contingency plan to conduct regular physical activity within a rural school experiencing consistently hot weather
		**Communications and strategic planning:** Processes explicitly related to or that guide the sustainment of a programme over time, e.g., through grant-writing, activities and engagement regarding sustainment, or marketing/communication plan focused on promoting the sustainment of an EBI	Dissemination of information and promotion of a school health-promoting programme through means of school newsletters, online platforms, school social media pages, or within local newspapers
**Characteristics of the Interventionist and Population**	• Provider/implementer characteristics • Implementation skills/expertise • Implementer attitudes • Implementer motivation • Population characteristics	**Implementer characteristics:** Implementer role self-efficacy, role clarity, commitment, and attitude	Perceived personal capability, motivation, and attitudes of the teachers delivering a health-promoting programme in schools
		**Implementer benefits and stressors:** Implementer benefits and stressors in role (including if paid or a volunteer)	Perceived personal benefit or stressors for teachers being involved in a health-promoting programme in schools, e.g., personal satisfaction knowing a programme will positively impact on students; or alternatively feeling overwhelmed with their own ability to deliver the programme given other school priorities
		**Implementer skills/expertise:** Prior knowledge, training, and motivation of the implementer	Perceived personal preparedness of teachers to adequately deliver the programme or intervention within schools, factoring in any previous training may have completed
		**Population characteristics:** Trust and medical mistrust, literacy, socioeconomic status, race/ethnicity, and experiences of stigma or discrimination among the target population	Appropriateness of the programme considering the SES of the population, e.g., rural schools. Further, this includes the appropriateness of programme resources and materials considering literacy levels of the target population
**Characteristics of the Intervention**	• Adaptability • Fit with population and context • Benefits/need • Burden/complexity • Trialability • Cost	**Adaptability of EBI/fidelity:** Degree to which an EBI can be tailored or refined to fit new settings or population needs, e.g., original guidelines or evidence vs. newer guidelines or evidence	Adaptability of a school-based health-promoting programme for teacher’s schedule and the school environment, e.g., adaptability of a programme to include contingency plans if materials and equipment are not available
		**Fit with context/population/organisation:** Fit of an EBI within a context, populations, and organisations as well as the perceived trust and medical mistrust of an EBI or source of evidence	Appropriateness of a health-promoting programme considering the context, culture, and population within schools to address an identified issue, e.g., childhood obesity; and inclusion of a credible source supporting the delivery of the programme, e.g., university or Area Health Service
		**Perceived benefits**: Perceived impact, evidence, cost, or relative advantage of an EBI	Value of a health-promoting programme within a school. Prioritisation of the programme over other competing interests, e.g., maths and English. School staff (principal and teachers) belief that the programme will be advantageous and the cost of the programme is appropriate
		**Perceived need**: Perceived need in the community or setting for an EBI or the topic it addresses	The value parents see in a school health-promoting program

^a^An exhaustive list of factors for each domain regarded as particularly important across multiple settings and contexts informed by the Integrated Sustainability Framework[9]

^b^Definitions for each factor regarded as particularly important specifically within schools and/or childcare services were informed by Shelton et al. [9] and collaboration with one of the developers of the Integrated Sustainability Framework (author RCS)

^c^Examples of how each factor could be applied within ECEC services predetermined by the research team in collaboration with one of the developers of the Integrated Sustainability Framework and outlined by Shoesmith et al.[15]

First, clear definitions for each domain deemed important to the ECEC setting were developed. These definitions were informed based on the framework, reviewed and iteratively updated by an expert panel, including one of the developers of the framework and experts in the ECEC setting, as well as experts in sustainability, measure development and psychometric evaluation. Second, an initial item pool of 87 items across the five framework domains was deductively generated [21] based on literature review evidence [15] and insight of eight content experts across the fields of implementation science, psychometric scale development, and education. Third, items were reduced through iterative discourse between the research team and the same content experts until consensus was reached on a comprehensive list of items (adequately covering all framework domains) without duplicates. Upon completion of this phase, the measure consisted of 42 items across five sustainability domain subscales: Outer Contextual Factors (5 items), Inner Contextual Factors (10 items), Processes (9 items), Characteristics of the Interventionist and Population (6 items), and Characteristics of the Intervention (12 items). The measure utilised a five-point Likert scale for each item, with response options: strongly agree; agree; neither agree nor disagree; disagree; strongly disagree. This was informed by other response scales of similar measures [47, 48] and recommendations of content experts in psychometric scale development.

#### Assessment of content validity and face validity

Content validity is the extent to which the items represent the constructs that a tool is designed to measure [21, 49]. Face validity is a component of content validity, and relates to the degree to which end-users deem the items as an appropriate representation of the target constructs [49]. An advisory group consisting of five content experts including two implementation scientists, two service delivery staff, and a Nominated Supervisor in an ECEC service, who were independent from those directly involved in generation of the initial item pool reviewed the initial measure. The advisory group reviewed the content and face validity of the measure by considering the following: (i) *“are the items of the measure relevant to what’s being measured?”*; (ii) *“does the measurement method seem useful for measuring the variable/construct?”*; and (iii) *“is the measure seemingly appropriate for capturing the variable/construct?”* The advisory group also reviewed each item to minimise misunderstanding and subsequent measurement error by enhancing item clarity, comprehensibility and relevance to the target population (ECEC service executives) [21]. Following Phase 1, the number of scale items reduced to 29.

### Phase 2: psychometric and pragmatic evaluation

Phase 2 involved a formal evaluation to assess the psychometric properties and pragmatic features of the IMPRESS-C according to the PAPERS criteria, which uses a six-point Likert scale ranging from − 1 (poor) to 4 (excellent) [24, 31]. The methods used are described below.

#### Ethical approval

We sought approval for this study from the Hunter New England Human Research Ethics Committee (no. 06/07/26/4.04 2019/ETH12353) and University of Newcastle Human Research Ethics Committee (no. H-2008–0343).

#### Design and sample

The psychometric and pragmatic properties of the scale were assessed via a national cross-sectional survey with executive staff (i.e., Nominated Supervisors, Service Directors, Service Owners and Room Leaders) from ECEC services across all six states and two territories of Australia. The preferred respondent from each service was the Nominated Supervisor. A sample size of 2,000 ECEC services was chosen to account for a 50% consent rate for the larger survey. The national sample was extracted from the publicly available Australian Children's Education & Care Quality Authority (ACECQA) register. All ECEC services in Australia are required to be listed in this register as part of national accreditation processes. To obtain a sample of 2,000 services, 2,050 services (oversampling to account for ineligible services) were randomly selected, and stratified by state, using a random number generator in Microsoft Excel.

#### Eligibility

Services were eligible if they were a centre-based ECEC service (preschools and long day cares) approved by ACECQA. Long day care services provide centre-based care for children from 6 weeks to under 6 years of age for eight or more hours per day. Preschools typically enrol children between 3 and 6 years of age and provide care for 6 to 8 h per day [50].

Services were ineligible if they were:

- A family day care service or provided only outside of school hours (OOSH) care;
- A Department of Education service (i.e., attached to a school due to falling under a different ethics jurisdiction);
- Temporarily closed according to ACECQA and based on telephone calls made from the research team;
- Operating only on Saturday and/or Sunday; or
- Providing care solely for children with special needs

#### Recruitment

Services were recruited using a staggered approach from August 2021 to April 2022. Each week approximately 250 services received an email inviting them to participate in the survey either online or via computer-assisted telephone interview (CATI). The order of contact was not randomised. A link directed services to the information statement (available for download), which then led to the online survey. Services were also mailed a hardcopy of the information statement, informing them that they will receive an email and a phone call inviting them to complete the survey. Approximately one week after the initial invitation, services which had not yet completed the online survey (including partial-completion) were first sent a reminder email and then a phone call by trained interviewers, inviting them to complete the survey via CATI.

#### Data collection

Surveys consisted of the 29-item IMPRESS-C, where respondents reported on the extent to which a number of factors influenced the continued delivery of an evidence-based program (targeting healthy eating or physical activity) at their service (a complete list of programs is illustrated in Additional file 1). Respondents were asked to complete the measure for one specific health promotion program. These programs were selected on the basis of systematic review findings [4, 5] and recommended for the setting to improve child healthy eating or physical activity. Each program of interest was selected based on previous responses of what programs were being implemented by the service. If the service was implementing multiple relevant programs, the program was assigned based on a hierarchy of programs. The hierarchy was initially determined based on the likelihood of services to implement each program across jurisdictions and was regularly updated throughout data collection to ensure an even distribution of responses to each of the included programs. The survey also asked for respondents’ demographics (current position, employment status, and highest level of relevant qualification completed that is related to ECEC employment); service characteristics (service type i.e., long day care or preschool, service hours of operation, age groups service cares for, number of full-time, part-time and casual educators working at the service, and number of children that attend the service on an average day); and service-level implementation of physical activity and healthy eating programs.

#### Statistical analysis

Statistical analyses were undertaken in R version 4.0.2 [51, 52]. An overview of the specific psychometric properties and the statistical analyses used are described below.

##### Item investigation

Item responses and response patterns were initially assessed to identify any items that were poorly responded to, and reviewed for possible exclusion. This included the distribution of responses for each item and percentage of respondents missing each item (missing included “don’t know” and “prefer not to say” responses). Items with more than 10% missing data or with more than 90% of responses occurring on only one of the response options were considered for potential exclusion. Polychoric correlations between all pairs of items were reviewed to help identify any possible redundancies in the items, with those with a correlation coefficient above 0.8 reviewed for possible exclusion by the research team [53].

##### Structural validity

As the dimensionality of the measure was based on an existing framework and we had a clear hypothesis of how the items of the scale should relate to one another [54], a confirmatory factor analysis (CFA) proposing a four-factor structure was selected. Diagonally weighted least squares was used as the estimation method due to the ordinal nature of the items [55, 56]. Parameter estimates were standardized with variances fixed at one. Missing responses, including those who answered ‘refused’ or ‘don’t know’ were imputed using a single imputation with predictive mean matching [57]. Respondents who missed all measure items were excluded from the analysis. An initial model assuming no correlation between factors was estimated and then revised to allow for such correlations, as it was reasonable to assume a relationship existed between the theoretical constructs. The following fit statistics and recommended criteria were used to assess the overall adequacy of the model:

- Standardized Root Square Residual (SRMR) < 0.08 [58, 59];
- Comparative Fit Index (CFI) > 0.95 [60];
- Root Mean Square Error of Approximation (RMSEA) < 0.07 [53, 58];
- Model Chi-squared *p*-value > 0.05 [61].

To reduce selection bias we pre-specified the criteria used to determine adequate fit indices, selecting those that have been recommended as they have been found to be most insensitive to the sample size, model misspecification and parameter estimates used [61]. Modification indices and factor loadings were examined and used to revise the CFA model to ensure the most parsimonious, adequate fitting and theoretically justifiable model was selected. Specifically, items with low factor loadings (< 0.40) or cross-loadings were examined, in consultation with the research team for removal or model amendments. Standardized factor loadings and their associated standard error, and *p*-values were reported.

##### Floor and ceiling effects

The percentage of respondents reporting the lowest and highest possible score for each domain were calculated. Domains where > 15% of respondents obtain the lowest (floor) or highest (ceiling) score were considered indicative of floor and ceiling effects [49].

##### Norms

Descriptive statistics for the final domains were calculated, including: median, quartiles one and three, minimum and maximum, mean, and standard deviation.

##### Internal consistency

Cronbach’s alpha was calculated for each domain, with values between 0.70 and 0.95 considered acceptable [49].

##### Concurrent validity

The correlation was used to examine the association between mean domain scores of the measure and the number of years a service reported to be delivering their specific health promotion program. Due to the non-linear relationship between the two measures, the Spearman correlation (Rho) was used. It was hypothesised that a moderate to high positive relationship between the measure domain scores and months of program delivery would be found. This was an assessment of concurrent validity as it assessed the agreement or correlation between two measures that theoretically should be tapping into similar constructs, administered at the same time [62, 63]. If these are true determinants of sustainability then they should be moderately or highly related to length of program delivery as this is a key indicator of sustainability. Based on the PAPERS scale, a correlation coefficient between 0.10 and 0.29 was considered ‘emerging’, 0.30 and 0.49 considered ‘adequate’, 0.50 and 0.69 ‘good’, and > 0.70 ‘excellent’ [24].

##### Known groups validity

Associations between the mean measure domain scores with characteristics hypothesised to differ were assessed using regression analysis. The known groups to compare included type of program (i.e., targeting physical activity or healthy eating), and number of full-time staff. Specifically, we hypothesised there would be a statistically significant difference between: i) program type for all four domains of the IMPRESS-C; and ii) number of full-time staff for all four domains of the measure. These characteristics have been found to have implications for sustainability as different programs may require varying levels of resources, support, and infrastructure to be maintained over time [64]; and a higher number of full-time staff can potentially lead to increased program capacity, more personalised attention to respondents, and greater adherence to program guidelines [43]. The mean domain scores were modelled individually as fixed effects. For program, a binomial distribution with a logistic link was used. The odds ratio (OR) or count ratio (CR) with corresponding 95% CI and *p*-values were presented. For full-time staff, a negative binomial distribution with a logistic link was used. The mean domain scores were modelled individually as fixed effects.

## Results

### Phase 1: item development, face and content validity

Of the initial 87-items, 58 were removed during item reduction processes. A total of 45 items were removed following expert feedback due to perceived duplication in item phrasing (17 items), or inadequacy of the item to cover the domain of interest (28 items). A further 13 items were removed following advisory group feedback due to difficulties in item interpretation (8 items) and limited perceived relevance or appropriateness for the ECEC setting (5 items). The final draft scale contained 29 items covering four domains of the Integrated Sustainability Framework – Outer Contextual Factors (4 items), Inner Contextual Factors (9 items), Processes (5 items) and Intervention Characteristics (11 items) (see Additional file 2 for complete item list). As a result of item development processes and assessment of face validity and content validity, factors relating to the Characteristics of the Interventionist and Population domain were deemed inappropriate to be answered by ECEC service executives as they do not have a comprehensive understanding of frontline intervention delivery. As such, this domain was removed from the measure.

### Phase 2: psychometric and pragmatic evaluation

Of the 1172 contacted services, 482 surveys were returned (*n =* 268 [57%] via telephone and *n =* 205 [43%] via online survey); the majority of which had full completion of measure items (*n =* 405 [84%]). Of the completed surveys with at least one response for measure items, 24 (5%) gave the same response for every item. Table 2 includes a breakdown of completed surveys by Australian states and territories. Surveys were completed by service executives (Nominated Supervisors *n =* 255 [54%], Service Directors *n =* 155 [33%], Room Leaders *n =* 11 [2.3%], and Service Owners *n =* 4 [0.8%]). Services cared for an average of 59 (SD = 31) children per day. For the health promotion program of interest, 241 (51%) respondents answered items based on a healthy eating program and 232 (49%) respondents answered based on a physical activity program. See Table 2 for a full description of respondent demographics and service characteristics.

**Table 2:** Demographic characteristics of participating early childcare services and service executives

Characteristics	n (%)
**Service level *n =* 473**	
**Service type**	
Long day care	430 (90.9%)
Preschool	43 (9.1%)
**State/Territory**	
New South Wales	199 (42%)
Queensland	95 (20%)
Victoria	86 (18%)
Western Australia	51 (11%)
South Australia	20 (4.2%)
Australian Capital Territory	11 (2.3%)
Tasmania	11 (2.3%)
**Region**	
Major cities of Australia	442 (93%)
Inner/outer regional Australia	31 (6.6%)
**Socio-economic Indexes for Areas (SEIFA)**	
Least disadvantaged	281 (59%)
Most disadvantaged	192 (41%)
**Service size (mean no. of children in service (SD))**	59 (31)
**Service executive level *n =* 473**	
**Position**	
Director	155 (33%)
Nominated supervisor	255 (54%)
Room leader	11 (2.3%)
Service owner	4 (0.8%)
Educator	9 (1.9%)
Other	39 (8.2%)
**Employment status (mean no per service (SD))**	
Full time staff	9 (8)
Part time staff^a^	7 (8)
Casual staff^a^	3 (4)
**Survey mode**	
Phone	268 (57%)
Online	205 (43%)
**Type of program measure completed on**	
Healthy eating	241 (51%)
Physical activity	232 (49%)

^a^Missing responses for these characteristics

#### Item investigation

Missing values were low for all 29 items, ranging from 0.8% to 3.7% (see Table 3). The full range of response options were used for 14 of the 29 items, although a left-hand skew was observed for all 29 items, with less than 5.9% of respondents utilising the lower end of the response scale, and most respondents answering towards the positive end of the scale. Polychoric correlation coefficients ranged from 0.03 to 0.77. No pairs of items recorded polychoric correlations above 0.8. However, of the 29 items, one item from the Inner Contextual Factors domain *“My service would be able to continue to deliver the program if there were changes to educators at our service”* possessed a high correlation (0.77) and when examined was considered conceptually similar to other items, therefore deemed appropriate to remove.

**Table 3:** Item-level information for the final 26-item IMPRESS-C measure

Domain and items	Missing n (%)	Standardised factor loading (SE)	*p-*value
**Domain: Outer Contextual Factors**			
My service governing body has a policy or guideline regarding the ongoing delivery of the program that my service follows. *(Note: A governing body refers to an educational department or authority e.g., Australian Children's Education & Care Quality Authority)*	14 (2.91%)	0.57 (0.04)	< 0.001
My service has external partnerships that provide support for the ongoing delivery of the program within my service *(Note: Examples of partnerships include national authorities, government agencies, councils and health organisations)*	16 (3.33%)	0.55 (0.04)	< 0.001
The program aligns with the priorities of my wider service community. *(Note: service community refers to administrators, teachers/educators, staff members, children, their parents/guardians and families directly involved with your service)*	14 (2.91%)	0.67 (0.04)	< 0.001
**Domain: Inner Contextual Factors**			
There are program champions in my service who positively influence others to continue to deliver the program. *(Note: a champion is a peer representative that drives the continued delivery of the program within the service*	14 (2.91%)	0.65 (0.03)	< 0.001
Management at my service support the ongoing delivery of the program	4 (0.80%)	0.83 (0.02)	< 0.001
Management at my service support the training of educators to enable the ongoing delivery of the program	4 (0.80%)	0.76 (0.02)	< 0.001
My service allocates sufficient space to support the ongoing delivery of the program	4 (0.80%)	0.81 (0.01)	< 0.001
My service has sufficient equipment to support the ongoing delivery of the program	6 (1.25%)	0.83 (0.01)	< 0.001
My service has sufficient funding to support the ongoing delivery of the program	11 (2.29%)	0.69 (0.02)	< 0.001
My service allocates sufficient time to support the ongoing delivery of the program	5 (1.04%)	0.81 (0.01)	< 0.001
My service would be able to continue to deliver the program if there was a change of leaders (e.g., management or champions) at our service	9 (1.87%)	0.69 (0.02)	< 0.001
**Domain: Processes**			
Educators at my service receive sufficient formal training to support the ongoing delivery of the program	7 (1.45%)	0.80 (0.02)	< 0.001
My service is involved with collecting information and providing feedback to educators regarding my service’s performance in the program. *(Note: This may be collected in the form of teacher/educator or child surveys, or room observations)*	11 (2.29%)	0.73 (0.02)	< 0.001
My service has a process to evaluate how well the program aligns with our priority areas and if it does not fit, it adapts the program as needed	8 (1.66%)	0.79 (0.02)	< 0.001
My service has a documented plan to continue the delivery of the program long-term	12 (2.50%)	0.75 (0.02)	< 0.001
My service promotes the ongoing delivery of the program to the wider service community e.g., through a website or newsletter. *(Note: service community refers to administrators, teachers/educators, staff members, children, their parents/guardians and families directly involved with your service)*	9 (1.87%)	0.71 (0.02)	< 0.001
**Domain: Characteristics of the Intervention**			
My service is able to adapt the program if resources/equipment are reduced	10 (2.08%)	0.71 (0.02)	< 0.001
My service is able to adapt the program to suit the service environment	8 (1.66%)	0.94 (0.01)	< 0.001
I can easily adapt the program to fit within my normal schedule	8 (1.66%)	0.85 (0.01)	< 0.001
The program is appropriate for my service, regardless of the socio-demographic region my service resides in	9 (1.87%)	0.87 (0.01)	< 0.001
The program is culturally appropriate for children at my service	7 (1.45%)	0.85 (0.01)	< 0.001
The program is widely accepted within my service by educators	7 (1.45%)	0.89 (0.01)	< 0.001
The program is easily delivered within my service	6 (1.25%)	0.91 (0.01)	< 0.001
I believe the program helps to improve the health of children at my service	6 (1.25%)	0.77 (0.02)	< 0.001
The cost to deliver the program in my service is acceptable	18 (3.75%)	0.68 (0.02)	< 0.001
Delivering the program is as important as other learning outcomes specified within the Early Years Learning Framework e.g., encouraging children to be confident and involved learners	8 (1.66%)	0.71 (0.02)	< 0.001

#### Structural validity

There were 473 participants included in the CFA model. An initial model (chi-sq = 1491, degrees of freedom [df] = 371) was run and the factor loadings and modification indices were examined for all items (see Additional file 3 for the model building process and model fit index comparisons, interfactor correlations for each CFA model, and item factor loadings). This model illustrated only one of the four model fit indices were within the pre-specified criteria for model adequacy (SRMR = 0.065; CFI = 0.831; and RMSEA = 0.080; *p*-value = < 0.001). One item from the Outer Contextual Factors domain *“The delivery of the program has influence on the business operations/income of my service (e.g., number of child enrolments)”*, exhibited a low factor loading of 0.33 and was therefore removed based on the pre-specified threshold (< 0.40). One item from the Characteristics of the Intervention domain *“I believe the program has been developed by a reputable organisation”* was removed based on the high modification indices and review due to cross-loadings with the Outer Contextual Factors domain (modification index = 101.9) and Processes domain (modification index = 64.3). A revised, four-factor model (chi-sq = 906, df = 293) was run which illustrated three of the four model fit indices were within the pre-specified criteria for model adequacy (SRMR = 0.056; CFI = 0.993; and RMSEA = 0.067) and indicated ‘good’ structural validity of the model according to the PAPERS scale [24]. However, the chi-square *p*-value was < 0.001, which was smaller than the pre-specified criteria (> 0.05). All factor loadings of the revised model were > 0.4 (see Table 3). This was the final CFA model, which resulted in 26 items being included in the final measure in the psychometric evaluation – Outer Contextual Factors (3 items), Inner Contextual Factors (8 items), Processes (5 items) and Intervention Characteristics (10 items). A one-factor model (chi-sq = 2008, df = 299) was run to compare and assess the suitability of the four-factor model. The fit indices of the one-factor model (SRMR = 0.079; CFI = 0.980; RMSEA = 0.110; *p*-value = < 0.001) indicated a worse fit than the four-factor model.

#### Floor and ceiling effects

No domains possessed > 15% of the responses at minimum nor maximum values, indicating a lack of floor and ceiling effects for all domains (Table 4).

**Table 4:** Domain-level results assessing the internal consistency, floor and ceiling effects, and norms

Domain	Standardised alpha	Floor % at min	Ceiling % at max	Mean (SD)	Median (Q1, Q3)	Minimum and maximum score
Outer Contextual Factors	0.53	0.21	7.40	3.93 (0.63)	4 (3.67, 4.33)	1.00 and 5.00
Inner Contextual Factors	0.89	0.21	7.40	4.09 (0.52)	4 (3.88, 4.50)	1.00 and 5.00
Processes	0.84	0.21	5.29	3.78 (0.65)	4 (3.40, 4.00)	1.00 and 5.00
Intervention Characteristics	0.92	0.00	9.73	4.19 (0.43)	4 (4.00, 4.50)	2.70 and 5.00

#### Norms

Domain scores ranged from a mean of 3.78 (SD = 0.65) (Processes domain) to 4.19 (SD = 0.43) (Outer Contextual Factors domain), and all domains possessed a median of four (see Table 4). The measure norms rated ‘good’ on the PAPERS scale [24].

#### Internal consistency

The Inner Contextual Factors, Processes, and Characteristics of the Intervention domains possessed ‘good’ internal consistency, with Cronbach’s alpha values between the pre-specified threshold of > 0.7 and < 0.95, ranging from 0.84 to 0.92 (see Table 4). The Outer Contextual Factors domain had a lower Cronbach’s alpha (α = 0.53).

#### Concurrent validity

There were statistically significant associations between the Outer Contextual Factors domain (*ρ* = 0.119, 95% CI: 0.02, 0.21, *p =* 0.017), the Inner Contextual Factors domain (*ρ* = 0.112, 95% CI: 0.01, 0.21, *p =* 0.024), and the number of years the program was delivered (Table 5). With correlation coefficients of between 0.10 and 0.29, this rated ‘emerging’ on the PAPERS scale [24].

**Table 5:** Domain-level results assessing concurrent validity and known groups validity

Domain	Concurrent validity		Known groups validity			
	**Variable: Years a centre has been delivering a health program**		**Variable: Type of program (physical activity or healthy eating)**		**Variable: Number of full-time staff**	
	**Spearman’s Rho [LCL, UCL]**	***p*-value**	**Odds ratio [LCL, UCL]**	***p*-value**	**Count ratio [LCL, UCL]**	***p*-value**
Outer Contextual Factors	0.119 [0.02, 0.21]	**0.017**	0.88 [0.66, 1.18]	0.398	1.04 [0.92, 1.18]	0.551
Inner Contextual Factors	0.112 [0.01, 0.21]	**0.024**	0.98 [0.69, 1.38]	0.893	1.04 [0.89, 1.21]	0.612
Processes	0.066 [-0.03, 0.16]	0.184	1.00 [0.76, 1.31]	0.980	1.11 [0.98, 1.25]	0.089
Intervention Characteristics	0.018 [-0.08, 0.11]	0.723	1.43 [0.94, 2.17]	0.098	0.97 [0.81, 1.17]	0.747

Significant* p-*values are bolded

#### Known groups validity

There were no statistically significant relationships between the measure domains and the number of full-time staff or type of program (Table 5). With two hypotheses tested but known-groups validity failing to be detected, this rated ‘poor’ on the PAPERS scale [24].

#### Pragmatic qualities

Based on the PAPERS pragmatic rating, the cost of the measure is ‘excellent’ as the measure is free and in the public domain. The Flesch-Kincaid readability score for the measure was 10.6, and therefore the language was deemed ‘good’ as it was between an 8th and 12th grade level (range: 8.0–12.99). The measure has ‘excellent’ assessor burden (ease of training) as it requires no training and has free automated administration. The 26-item measure has ‘good’ length with > 10 items but ≤ 50 items. However, scoring requires manual calculation and additional inspection of response patterns or subscales, and no instructions for handling missing data are provided, which is a rating of ‘emerging’ on the PAPERS scale for assessor burden (easy to interpret) [24].

## Discussion

This study aimed to develop and evaluate the psychometric and pragmatic properties of the IMPRESS-C, the first known measure of sustainability determinants specific to the ECEC setting. This advances emerging work on the measurement of determinants influential to EBI sustainment in community settings [20, 27] by offering a theory-based measure informed by the Integrated Sustainability Framework [9]. A comprehensive development and evaluation process based on best practice guidelines [31, 46] was undertaken which resulted in a measure with strong content and face validity. The final 26-item IMPRESS-C was evaluated using a large national sample size for psychometric and pragmatic testing (> 350 competed surveys) [21, 65], and illustrated ‘good’ structural validity, ‘good’ internal consistency, ‘emerging’ concurrent validity, ‘poor’ known groups validity, ‘good’ norms, and ‘good’ pragmatic properties (i.e., cost, readability, length, and assessor burden – ease of training). The measure provides a novel assessment of the factors that may contribute to the sustainability of EBIs within ECEC settings from the executive-level perspective – important information to help guide policymakers and practitioners in the accurate development of strategies to target identified determinants and support EBI sustainability. However, further refinement of the measure and development of additional measures of sustainability determinants tailored to different end user perspectives (e.g., service educators [program implementers]) is needed to achieve a more holistic and comprehensive understanding of such factors.

The measure was developed using a rigorous and iterative approach based on gold standard measure development procedures [24, 46] with extensive input from a range of experts. Despite this rigorous process, we were limited by time and resource constraints and were unable to follow all aspects of the gold standard procedures, including extensive pre-testing and cognitive interviews with the target population. Future measure development studies in this setting should strive to conduct cognitive interviews with a separate sample of the target population to provide granular feedback on item comprehension, enhance response processes and ensure a more robust assessment of face and content validity [21, 66]. Further, this measure should be complemented with additional measures of sustainability determinants at the level of implementer or frontline intervention delivery to obtain a full range of perspectives within this specific setting to assess additional constructs important to sustainability e.g., motivation, self-efficacy, skill acquisition, and perceived individual benefits and stressors [20, 25]. This would also facilitate a more comprehensive and accurate understanding of the determinants important to the sustainability of EBIs in the ECEC setting and inform the development and tailoring of strategies to support intervention sustainment.

The measure illustrated ‘good’ internal consistency according to the PAPERS scale [24], with Cronbach’s alpha values for three of the four domains falling between the pre-specified threshold (Inner Contextual Factors, Processes and Intervention Characteristics). This is indicative that measurement reliability for these three domains is high. However, Outer Contextual Factors possessed a lower Cronbach’s alpha value which may be attributed to the lower number of items covered under that domain in comparison to the others [67]. To improve this for future research, focus should be placed on the creation, refinement and testing of additional items within this domain to yield higher internal consistency, provided such items remain relevant to external or Outer Contextual Factors.

Assessment of structural validity found three of the four model fit indices were within the pre-specified criteria indicating ‘good’ structural validity of the model according to the PAPERS scale [24]. This demonstrates that the measure accurately reflects the underlying structure or constructs it intends to assess (i.e., domains and constructs of the Integrated Sustainability Framework). However, we failed to meet the chi-square *p*-value criteria of > 0.05. The chi-square test is a difficult criteria to meet and is quite sensitive particularly when applied to moderate to large sample sizes, therefore, it is more likely to detect small differences that may not have as big an impact. This solidifies the need to have multiple indices to assess structural validity against. Further, although the measure was theoretically informed by the Integrated Sustainability Framework [9] and included constructs that reflect the main determinants found to influence EBI sustainability from the perspective of the service executive, we only included four of the five framework domains. Thus, it does not capture sustainability determinants associated with frontline intervention delivery and implementation. Again, highlighting the need for additional measures to assess these characteristics from the implementer perspective [20, 25].

For the assessment of known-groups validity, we found no evidence to support our initial hypotheses i.e., no statistically significant relationships between the framework domains and the number of full-time staff, nor the type of program. Potential reasons for our hypotheses not being supported could be a lack of difference in determinants between nutrition and physical activity programs as these are both important and related health behaviours often targeted simultaneously in EBI delivery [29]. Therefore, it is possible they possess the same or similar determinants for sustainability. It is important to assess the ability of the measure to accurately discriminate between groups that are expected to have distinct levels or characteristics on the construct of interest [21]. Based on these findings, it may be necessary to revisit the hypotheses, to obtain more robust evidence for known groups validity for the measure. However, given the lack of empirical evidence available that informs where the differences may lie, it is difficult to determine what known groups may exist at this time.

Examination of concurrent validity found a statistically significant relationship between the Outer Contextual Factors domain (*p =* 0.017) (e.g., external partnerships, socio-political support), the Inner Contextual Factors domain (*p =* 0.024) (e.g., organisational readiness and resources, executive leadership and support, workforce turnover), and the number of years the program was delivered. This exhibited a strong positive relationship between these domains of sustainability determinants and the length of program delivery, which is an expected finding given the length of program delivery is a primary indicator of its sustainment as intended (i.e., the longer a program is delivered, the longer it is sustained) [68]. Given the limited research in this area and lack of gold standard measurement into sustainability and sustainability determinants, there are limited other measures and constructs that could be confidently used to assess additional forms of validity for the IMPRESS-C. As the field progresses and researchers become more aware of determinants impacting intervention sustainability, we recommend future research continues to assess and improve the validity of the IMPRESS-C measure.

Although responsiveness was unable to be assessed, examination of floor and ceiling effects, which are indicators of this, was conducted to ensure the potential for the IMPRESS-C to detect change [49]. No domains possessed floor or ceiling effects, with < 15% of the responses at minimum and maximum values. However, only 5.9% of respondents answered the low end of the response scale. To mitigate this, strategies are needed such as reviewing and testing different response scales (i.e., with varying response options and number of options); and increasing item difficulty so that it better reflects the higher end of the response scale to be captured, potentially making the measure more sensitive to change [69].

The IMPRESS-C possessed an ‘excellent’ PAPERS rating for cost by ensuring the measure is in the public domain, a ‘good’ language rating by ensuring the readability of the measure was between an 8th and 12th grade level, ‘good’ length by ensuring the measure possessed < 50 items, and ‘excellent’ ease of training as it required no training and had free automated administration [24]. These qualities provide a highly pragmatic and user-friendly measurement tool for researchers to capture the priority executive-level determinants impacting on EBI sustainment within the ECEC setting [70]. Despite the good pragmatic qualities of the measure, further refinement to reduce assessor burden could be achieved by providing clear cut-off scores with value labels, instructions for handling missing data, and automated calculation of measure scores.

## Conclusion

The IMPRESS-C possesses good psychometric and pragmatic qualities for assessing executive-level perceptions of determinants influencing sustainment of public health interventions in the ECEC setting. Future efforts should be directed at refining this measure to further improve its psychometric and pragmatic properties, and complementing this measure with a valid and reliable measure of sustainability determinants targeting frontline intervention delivery staff. This would enable understanding of a range of perspectives among key end-users responsible for the delivery and governance of EBIs in ECEC settings and help inform a comprehensive and tailored approach to developing strategies supporting EBI sustainment within the setting.

### Supplementary Information

Supplementary Material 1.

Supplementary Material 2.

Supplementary Material 3.

## Data Availability

Data and materials are available from the corresponding author on reasonable request.

## Abbreviations

ACECQA: Australian Children's Education & Care Quality Authority
CATI: Computer-assisted telephone interview
CFA: Confirmatory factor analysis
CFI: Comparative Fit Index
COSMIN: COnsensus-based Standards for the selection of health status Measurement INstruments
EBI: Evidence-based intervention
ECEC: Early childhood education and care
IMPRESS-C: Integrated Measure of PRogram Element SuStainability in Childcare Settings
PAPERS: Psychometric and Pragmatic Evidence Rating Scale
OOSH: Outside of school hours
RMSEA: Root Mean Square Error of Approximation
SRMR: Standardized Root Square Residual
