# Supplementary material for: Integrated Measure of PRogram Element SuStainability in Childcare Settings (IMPRESS-C): development and psychometric evaluation of a measure of sustainability determinants in the early childhood education and care setting
Source: Implement Sci. 2024 Jun 20;19:41. doi: 10.1186/s13012-024-01372-w (PMC11188265; doi:10.1186/s13012-024-01372-w)
Supplement: Supplementary file 1 — Supplementary Material 1. [file 13012_2024_1372_MOESM1_ESM.docx]

**Additional file 1.** Program names and definitions

| **Program** | **Definition of program** |
| --- | --- |
| **Physical activity programs** | |
| Providing opportunities for child physical activity | This means providing children aged 3-5 with sufficient opportunity for physical activity every day. When thinking about this, we mean taking specific actions to schedule at least 180 minutes each day where children are able to be physical active, such as through active free play and educator-led structured physical activity. This includes engaging children in moderate-to-vigorous physical activity such as running, dancing or jumping. |
| Providing educator training opportunities and ongoing support | This means providing training and support to educators in child physical activity at least annually. When thinking about this, we mean taking specific actions to provide or support face-to-face or online training. This may be through an internal or external organisation, *as well as* providing ongoing support and resources, such as additional group meetings, refresher training or onsite visits by the training provider. |
| Delivering teacher-led physical activity | This means including educator-led, structured physical activity in the daily routine, specifically to increase childrens’ physical activity and teach complex motor skills, such as catching, jumping and kicking. |
| Engaging families in child physical activity | This means engaging families in their child’s physical activity. When thinking about this, we mean taking specific actions to provide education to families about children’s physical activity through workshops or newsletters at least once per year, or asking families for feedback on physical activity standards in care. |
| Providing portable playground equipment | This means providing children with access to suitable portable play equipment every day. This includes making sure that there is enough portable play equipment, such as balls, hula hoops or tricycles, for children to share. |
| Having a physical activity policy | This means having a comprehensive physical activity policy for children. When thinking about this, we mean taking specific actions to implement a physical activity policy which includes reference to the Australian 24-Hour Movement Guidelines for the Early Years (Birth to 5 Years), active play learning experiences, educator role modelling, *and* both child- and educator-led play. |
| **Healthy eating programs** | |
| Making healthy menu modifications | This means implementing strategies to ensure the foods and beverage provided meet state or national nutrition guidelines (e.g. Australian Dietary Guidelines). When thinking about this, we mean having menu planners/service cooks trained in nutrition and supporting them with mentoring and/or networking opportunities, written resources, nutrition guidelines or linking them with external experts. |
| Providing families with lunchbox guidelines | This means providing resources to families to improve the packing of child lunchboxes. When thinking about this program, we mean taking specific actions to provide parents with lunchbox guidelines which refer to the Australian Guide to Healthy Eating *and* providing feedback regularly (at least 4 times per year) to families around the packing of healthy lunchboxes when foods do not meet guidelines. |
| Supporting educators around child healthy eating | This means supporting staff to implement strategies which improve children’s healthy eating. This includes training educators around ways to promote healthy eating for children, providing staff with written resources, and monitoring educators’ performance and providing feedback on their implementation of healthy eating strategies for children. |
| Providing healthy eating education for children | This includes providing children with any of the following: planned healthy eating education lessons; interactive healthy eating activities such as cooking; exposure to different vegetables; and, play-based healthy eating activities such reading healthy eating books or using puppets to talk about healthy eating at least weekly. |
| Role-modelling healthy eating to children | This means peer and teacher role-modelling of healthy eating during each meal. When thinking about this, we mean taking specific actions to promote peer role-modelling of eating healthier foods, and staff only eating healthier foods with children at mealtimes. |
| Encouraging healthy drink choices | This means encouraging healthy and age-appropriate drinks for children every day. This includes making sure that sugar-sweetened beverages such as flavoured milks or juice are not available to children while in care *and* having a policy which supports having water as a drink for children aged 3-5 years.” |
