# Supplementary material for: Integrated Measure of PRogram Element SuStainability in Childcare Settings (IMPRESS-C): development and psychometric evaluation of a measure of sustainability determinants in the early childhood education and care setting
Source: Implement Sci. 2024 Jun 20;19:41. doi: 10.1186/s13012-024-01372-w (PMC11188265; doi:10.1186/s13012-024-01372-w)
Supplement: Supplementary file 2 — Supplementary Material 2. [file 13012_2024_1372_MOESM2_ESM.docx]

**Additional file 2.** Complete item list for the original IMPRESS-C

| **Original IMPRESS-C scale items following measure development** | **Removed during psychometric evaluation** |
| --- | --- |
| **Domain: Outer contextual factors** | |
| 1. My service governing body has a policy or guideline regarding the ongoing delivery of the program that my service follows. *(Note: A governing body refers to an educational department or authority e.g., Australian Children's Education & Care Quality Authority).* | No |
| 2. The delivery of the program has influence on the business operations/income of my service *(e.g., number of child enrolments).* | Yes |
| 3. My service has external partnerships that provide support for the ongoing delivery of the program within my service.  (Note: Examples of partnerships include national authorities, government agencies, councils and health organisations). | No |
| 4. The program aligns with the priorities of my wider service community. *(Note: service community refers to administrators, teachers/educators, staff members, children, their parents/guardians and families directly involved with your service).* | No |
| **Domain: Inner contextual factors** | |
| 5. There are program champions in my service who positively influence others to continue to deliver the program. *(Note: a champion is a peer representative that drives the continued delivery of the program within the service.* | No |
| 6. Management at my service support the ongoing delivery of the program. | No |
| 7. Management at my service support the training of educators to enable the ongoing delivery of the program. | No |
| 8. My service allocates sufficient space to support the ongoing delivery of the program. | No |
| 9. My service has sufficient equipment to support the ongoing delivery of the program. | No |
| 10. My service has sufficient funding to support the ongoing delivery of the program. | No |
| 11. My service allocates sufficient time to support the ongoing delivery of the program. | No |
| 12. My service would be able to continue to deliver the program if there was a change of leaders (e.g., management or champions) at our service. | No |
| 13. My service would be able to continue to deliver the program if there were changes to educators at our service. | Yes |
| **Domain: Processes** | |
| 14. Educators at my service receive sufficient formal training to support the ongoing delivery of the program. | No |
| 15. My service is involved with collecting information and providing feedback to educators regarding my service’s performance in the program. *(Note: This may be collected in the form of teacher/educator or child surveys, or room observations).* | No |
| 16. My service has a process to evaluate how well the program aligns with our priority areas and if it does not fit, it adapts the program as needed. | No |
| 17. My service has a documented plan to continue the delivery of the program long-term. | No |
| 18. My service promotes the ongoing delivery of the program to the wider service community e.g., through a website or newsletter. *(Note: service community refers to administrators, teachers/educators, staff members, children, their parents/guardians and families directly involved with your service).* | No |
| **Domain: Characteristics of the intervention** | |
| 19. My service is able to adapt the program if resources/equipment are reduced. | No |
| 20. My service is able to adapt the program to suit the service environment. | No |
| 21. I can easily adapt the program to fit within my normal schedule. | No |
| 22. The program is appropriate for my service, regardless of the socio-demographic region my service resides in. | No |
| 23. The program is culturally appropriate for children at my service. | No |
| 24. I believe the program has been developed by a reputable organisation. | Yes |
| 25. The program is widely accepted within my service by educators. | No |
| 26. The program is easily delivered within my service. | No |
| 27. I believe the program helps to improve the health of children at my service. | No |
| 28. The cost to deliver the program in my service is acceptable. | No |
| 29. Delivering the program is as important as other learning outcomes specified within the Early Years Learning Framework e.g., encouraging children to be confident and involved learners. | No |
