# Supplementary material for: Integrated Measure of PRogram Element SuStainability in Childcare Settings (IMPRESS-C): development and psychometric evaluation of a measure of sustainability determinants in the early childhood education and care setting
Source: Implement Sci. 2024 Jun 20;19:41. doi: 10.1186/s13012-024-01372-w (PMC11188265; doi:10.1186/s13012-024-01372-w)
Supplement: Supplementary file 3 — Supplementary Material 3. [file 13012_2024_1372_MOESM3_ESM.docx]

**ADDITIONAL FILE 3. Model building process, interfactor correlations, and item factor loadings**

**Additional file 3a. Model building process and CFA model fit index comparisons:**

- Model 1: Likert scale treated as continuous. Covariance fixed at 0. Contained four factors.
- Model 2: Likert scale treated as continuous. Covariance not fixed. Contained four factors.
- Model 3: Likert scale treated as ordinal. Covariance not fixed. Contained four factors. Imputed dataset.
- Model 4: Likert scale treated as ordinal. Covariance not fixed. Contained four factors. Imputed dataset. Removed items: *“The delivery of the program has influence on the business operations/income of my service”* and *“I believe the program has been developed by a reputable organisation”.*
- Model 5: Likert scale treated as ordinal. Covariance not fixed. Contained one factor. Imputed dataset. Removed items: *“The delivery of the program has influence on the business operations/income of my service”* and *“I believe the program has been developed by a reputable organisation”.*

**Table 1.** Model fit indices of tested models

| **Model** | **Chi sq** | **df** | **CFI** | **RMSEA** | **SRMR** |
| --- | --- | --- | --- | --- | --- |
| Model 1 | 2180.692 | 377 | 0.728 | 0.100 | 0.249 |
| Model 2 | 1491.892 | 371 | 0.831 | 0.080 | 0.065 |
| Model 3 | 1675.631 | 371 | 0.987 | 0.086 | 0.066 |
| **Model 4*** | **906.073** | **293** | **0.993** | **0.067** | **0.056** |
| Model 5 | 2008.190 | 299 | 0.980 | 0.110 | 0.079 |

**Model 4 is highlighted and bolded as this was the final model used.*

**Table 2.** Nested model comparison between Model 1 and Model 2

| **Chi sq difference test** | | **Model difference (model 2 - model 1)** | | | |
| --- | --- | --- | --- | --- | --- |
| **Chi sq** | **p-value** | **df** | **CFI** | **RMSEA** | **SRMR** |
| 386.633 | <0.001 | 6 | -0.103 | 0.021 | 0.184 |

**Table 3.** Nested model comparison between Model 5 and Model 4

| **Chi sq difference test** | | **Model difference (model 5 - model 4)** | | | |
| --- | --- | --- | --- | --- | --- |
| **Chi sq** | **p-value** | **df** | **CFI** | **RMSEA** | **SRMR** |
| 530.916 | <0.001 | 6 | -0.013 | 0.043 | 0.023 |

**Additional file 3b**. Interfactor correlation matrices for each confirmatory factor analysis model

**Table 4.** Interfactor correlation matrix for Model 2

|  | **Outer contextual factors** | **Inner contextual factors** | **Processes** | **Characteristics of the intervention** |
| --- | --- | --- | --- | --- |
| **Outer contextual factors** | 1.000 |  |  |  |
| **Inner contextual factors** | 0.594 | 1.000 |  |  |
| **Processes** | 0.707 | 0.629 | 1.000 |  |
| **Characteristics of the intervention** | 0.542 | 0.805 | 0.609 | 1.000 |

**Table 5.** Interfactor correlation matrix for Model 3

|  | **Outer contextual factors** | **Inner contextual factors** | **Processes** | **Characteristics of the intervention** |
| --- | --- | --- | --- | --- |
| **Outer contextual factors** | 1.000 |  |  |  |
| **Inner contextual factors** | 0.598 | 1.000 |  |  |
| **Processes** | 0.738 | 0.690 | 1.000 |  |
| **Characteristics of the intervention** | 0.626 | 0.816 | 0.706 | 1.000 |

**Table 6.** Interfactor correlation matrix for Model 4

|  | **Outer contextual factors** | **Inner contextual factors** | **Processes** | **Characteristics of the intervention** |
| --- | --- | --- | --- | --- |
| **Outer contextual factors** | 1.000 |  |  |  |
| **Inner contextual factors** | 0.692 | 1.000 |  |  |
| **Processes** | 0.762 | 0.707 | 1.000 |  |
| **Characteristics of the intervention** | 0.644 | 0.835 | 0.693 | 1.000 |

*Note: The model factor correlations for Model 1 weren’t included as the covariance was fixed at 0, or for Model 5 as this model only included one factor.*

**Additional file 3c.** Item factor loadings for the Model 1 and Model 3

**Table 7.** Item factor loadings for Model 1

| **Domain and items** | **Standardised Factor Loading (SE)** | **p-value** |
| --- | --- | --- |
| **Domain: Outer contextual factors** | | |
| My service governing body has a policy or guideline regarding the ongoing delivery of the program that my service follows. *(Note: A governing body refers to an educational department or authority e.g., Australian Children's Education & Care Quality Authority).* | 0.60 (0.04) | <0.001 |
| The delivery of the program has influence on the business operations/income of my service *(e.g., number of child enrolments).* | 0.35 (0.04) | <0.001 |
| My service has external partnerships that provide support for the ongoing delivery of the program within my service. (Note: Examples of partnerships include national authorities, government agencies, councils and health organisations). | 0.63 (0.04) | <0.001 |
| The program aligns with the priorities of my wider service community. *(Note: service community refers to administrators, teachers/educators, staff members, children, their parents/guardians and families directly involved with your service).* | 0.73 (0.04) | <0.001 |
| **Domain: Inner contextual factors** | | |
| There are program champions in my service who positively influence others to continue to deliver the program. *(Note: a champion is a peer representative that drives the continued delivery of the program within the service.* | 0.67 (0.03) | <0.001 |
| Management at my service support the ongoing delivery of the program. | 0.82 (0.02) | <0.001 |
| Management at my service support the training of educators to enable the ongoing delivery of the program. | 0.77 (0.02) | <0.001 |
| My service allocates sufficient space to support the ongoing delivery of the program. | 0.81 (0.02) | <0.001 |
| My service has sufficient equipment to support the ongoing delivery of the program. | 0.82 (0.02) | <0.001 |
| My service has sufficient funding to support the ongoing delivery of the program. | 0.70 (0.02) | <0.001 |
| My service allocates sufficient time to support the ongoing delivery of the program. | 0.80 (0.02) | <0.001 |
| My service would be able to continue to deliver the program if there was a change of leaders (e.g., management or champions) at our service. | 0.79 (0.02) | <0.001 |
| My service would be able to continue to deliver the program if there were changes to educators at our service. | 0.75 (0.02) | <0.001 |
| **Domain: Processes** | | |
| Educators at my service receive sufficient formal training to support the ongoing delivery of the program. | 0.82 (0.02) | <0.001 |
| My service is involved with collecting information and providing feedback to educators regarding my service’s performance in the program. *(Note: This may be collected in the form of teacher/educator or child surveys, or room observations).* | 0.73 (0.02) | <0.001 |
| My service has a process to evaluate how well the program aligns with our priority areas and if it does not fit, it adapts the program as needed. | 0.79 (0.02) | <0.001 |
| My service has a documented plan to continue the delivery of the program long-term. | 0.77 (0.02) | <0.001 |
| My service promotes the ongoing delivery of the program to the wider service community e.g., through a website or newsletter. *(Note: service community refers to administrators, teachers/educators, staff members, children, their parents/guardians and families directly involved with your service).* | 0.71 (0.02) | <0.001 |
| **Domain: Characteristics of the intervention** | | |
| My service is able to adapt the program if resources/equipment are reduced. | 0.71 (0.02) | <0.001 |
| My service is able to adapt the program to suit the service environment. | 0.93 (0.01) | <0.001 |
| I can easily adapt the program to fit within my normal schedule. | 0.86 (0.01) | <0.001 |
| The program is appropriate for my service, regardless of the socio-demographic region my service resides in. | 0.87 (0.01) | <0.001 |
| The program is culturally appropriate for children at my service. | 0.85 (0.01) | <0.001 |
| I believe the program has been developed by a reputable organisation. | 0.65 (0.02) | <0.001 |
| The program is widely accepted within my service by educators. | 0.89 (0.01) | <0.001 |
| The program is easily delivered within my service. | 0.91 (0.01) | <0.001 |
| I believe the program helps to improve the health of children at my service. | 0.76 (0.02) | <0.001 |
| The cost to deliver the program in my service is acceptable. | 0.69 (0.02) | <0.001 |
| Delivering the program is as important as other learning outcomes specified within the Early Years Learning Framework e.g., encouraging children to be confident and involved learners. | 0.69 (0.02) | <0.001 |

**Table 7.** Item factor loadings for Model 3

| **Domain and items** | **Standardised Factor Loading (SE)** | **p-value** |
| --- | --- | --- |
| **Domain: Outer contextual factors** | | |
| My service governing body has a policy or guideline regarding the ongoing delivery of the program that my service follows. *(Note: A governing body refers to an educational department or authority e.g., Australian Children's Education & Care Quality Authority).* | 0.61 (0.04) | <0.001 |
| The delivery of the program has influence on the business operations/income of my service *(e.g., number of child enrolments).* | **0.33 (0.04)** | <0.001 |
| My service has external partnerships that provide support for the ongoing delivery of the program within my service. (Note: Examples of partnerships include national authorities, government agencies, councils and health organisations). | 0.60 (0.04) | <0.001 |
| The program aligns with the priorities of my wider service community. *(Note: service community refers to administrators, teachers/educators, staff members, children, their parents/guardians and families directly involved with your service).* | 0.70 (0.04) | <0.001 |
| **Domain: Inner contextual factors** | | |
| There are program champions in my service who positively influence others to continue to deliver the program. *(Note: a champion is a peer representative that drives the continued delivery of the program within the service.* | 0.65 (0.03) | <0.001 |
| Management at my service support the ongoing delivery of the program. | 0.82 (0.02) | <0.001 |
| Management at my service support the training of educators to enable the ongoing delivery of the program. | 0.76 (0.02) | <0.001 |
| My service allocates sufficient space to support the ongoing delivery of the program. | 0.82 (0.02) | <0.001 |
| My service has sufficient equipment to support the ongoing delivery of the program. | 0.83 (0.02) | <0.001 |
| My service has sufficient funding to support the ongoing delivery of the program. | 0.70 (0.02) | <0.001 |
| My service allocates sufficient time to support the ongoing delivery of the program. | 0.81 (0.02) | <0.001 |
| My service would be able to continue to deliver the program if there was a change of leaders (e.g., management or champions) at our service. | 0.79 (0.02) | <0.001 |
| My service would be able to continue to deliver the program if there were changes to educators at our service. | 0.75 (0.02) | <0.001 |
| **Domain: Processes** | | |
| Educators at my service receive sufficient formal training to support the ongoing delivery of the program. | 0.81 (0.02) | <0.001 |
| My service is involved with collecting information and providing feedback to educators regarding my service’s performance in the program. *(Note: This may be collected in the form of teacher/educator or child surveys, or room observations).* | 0.73 (0.02) | <0.001 |
| My service has a process to evaluate how well the program aligns with our priority areas and if it does not fit, it adapts the program as needed. | 0.78 (0.02) | <0.001 |
| My service has a documented plan to continue the delivery of the program long-term. | 0.75 (0.02) | <0.001 |
| My service promotes the ongoing delivery of the program to the wider service community e.g., through a website or newsletter. *(Note: service community refers to administrators, teachers/educators, staff members, children, their parents/guardians and families directly involved with your service).* | 0.71 (0.02) | <0.001 |
| **Domain: Characteristics of the intervention** | | |
| My service is able to adapt the program if resources/equipment are reduced. | 0.71 (0.02) | <0.001 |
| My service is able to adapt the program to suit the service environment. | 0.93 (0.01) | <0.001 |
| I can easily adapt the program to fit within my normal schedule. | 0.85 (0.01) | <0.001 |
| The program is appropriate for my service, regardless of the socio-demographic region my service resides in. | 0.87 (0.01) | <0.001 |
| The program is culturally appropriate for children at my service. | 0.85 (0.01) | <0.001 |
| I believe the program has been developed by a reputable organisation. | 0.62 (0.02) | <0.001 |
| The program is widely accepted within my service by educators. | 0.89 (0.01) | <0.001 |
| The program is easily delivered within my service. | 0.91 (0.01) | <0.001 |
| I believe the program helps to improve the health of children at my service. | 0.76 (0.02) | <0.001 |
| The cost to deliver the program in my service is acceptable. | 0.68 (0.02) | <0.001 |
| Delivering the program is as important as other learning outcomes specified within the Early Years Learning Framework e.g., encouraging children to be confident and involved learners. | 0.71 (0.02) | <0.001 |

*Note. The bolded standardised factor loading (SE) represented the item that was removed due to a low factor loading (<0.40).*
